# Novel probiotic *Lactobacillus helveticus* WIS02 alleviates diabetes through multi-pronged regulation of glycolipid metabolism, pancreatic protection and gut microbiota remodeling

**DOI:** 10.3389/fmicb.2025.1735605

**Published:** 2026-01-21

**Authors:** Shuang Guo, Yuying Li, Yanan Yang, Yuexiao Jiang, Yufeng Wang, Ying Cao, Yunfeng Duan, Chongming Wu

**Affiliations:** 1 School of Chinese Materia Medica, Tianjin University of Traditional Chinese Medicine, Tianjin, China; 2 PKUMed-Wisbiom Joint Laboratory for Human Microbiome Research, Beijing, China; 3 State Key Laboratory of Chinese Medicine Modernization, Tianjin, China; 4 Tianjin Key Laboratory of Therapeutic Substance of Traditional Chinese Medicine, Tianjin, China

**Keywords:** diabetes mellitus, gut microbiota, hypoglycemic effect, *Lactobacillus helveticus* WIS02, probiotic

## Abstract

**Background:**

Diabetes mellitus poses a global health burden with limited therapies derived from breast milk targeting its systemic complications. Probiotics like *Lactobacillus helveticus* show metabolic benefits, but strain-specific efficacy and mechanisms remain unclear.

**Methods:**

This study evaluated *L. helveticus* WIS02’s therapeutic potential in streptozotocin (STZ)-induced diabetic mice, focusing on glycemic control, tissue protection, and gut microbiota modulation.

**Results:**

We found pre-administration of WIS02 for 7 days reduced the final fasting blood glucose by 66.0% and improved oral glucose tolerance (AUC decreased by 51.6%). Additionally, WIS02 significantly mitigated dyslipidemia (TC decreased by 23.6%, TG decreased by 39.5%) and alleviated tissue damage to the liver, pancreatic, and colon tissues. Microbiota analysis revealed that WIS02 treatment significantly altered the gut microbiota structure, increasing the proportion of beneficial bacteria, particularly *Akkermansia muciniphila* and *Parabacteroides distasonis.* Correlation analysis between microbiota abundance and physiological indicators showed that *Parabacteroides distasonis*, *Oscillibacter valericigenes*, and *Akkermansia muciniphila* were significantly negatively correlated with blood glucose and lipid levels, while positively correlated with insulin and hepatic glycogen levels.

**Conclusion:**

*L. helveticus* WIS02 exhibits multi-targeted efficacy against diabetes by regulating glycolipid metabolism, protecting tissues, and remodeling gut microbiota. These findings highlight its potential as a novel probiotic for metabolic disorder management, warranting clinical translation.

## 1 Introduction

Diabetes mellitus (DM) represents a pressing global public health crisis characterized by chronic hyperglycemia stemming from impaired insulin secretion or sensitivity. Classified primarily into type 1 diabetes (T1D), type 2 diabetes (T2D), and gestational diabetes (GDM), among others, its prevalence has surged to approximately 10.5% of the global population, with diabetes-related cardiovascular and renal complications driving rising mortality rates (Li et al., 2020; Hossain et al., 2024; Mokdad et al., 2024). Conventional therapies, while foundational, fail to address the multifaceted nature of DM pathogenesis. Metformin, the first-line agent for T2D, reduces hepatic gluconeogenesis but imposes renal metabolic stress, with 23% of long-term users developing drug-induced renal impairment (Bailey, 2024). Sulfonylureas and insulin, though effective, carry risks of hypoglycemia and weight gain, complicating adherence in elderly or obese populations (Bailey and Mezitis, 1990). The lifelong treatment burden—compounded by strict dietary restrictions and frequent monitoring—leads to 40% non-compliance rates, further worsening clinical outcomes. These limitations underscore the critical need for novel strategies that target underlying mechanisms beyond glycemic control.

The gut microbiota has emerged as a pivotal regulator of human health (Duan et al., 2022; Dong et al., 2023; Yang et al., 2024), as well as a therapeutic target of several drugs (Li et al., 2023; Yang et al., 2023; Su et al., 2024; Zou et al., 2024). Probiotics, prebiotics, and fecal microbiota transplantation (FMT) can modulate microbial ecology to improve metabolic homeostasis (Chen et al., 2024; Wang et al., 2024). Probiotics replenish beneficial bacteria to suppress pathogens and restore flora balance (Zhou et al., 2025), while prebiotics enhance commensal growth through nutritional support (Iatcu et al., 2024); FMT achieves microbial reconstitution via transfer of healthy donor microbiota (Gómez-Pérez et al., 2024). *Akkermansia muciniphila* administration, for example, reduces HbA1c in T2D patients (Su et al., 2024), while *Bifidobacterium animalis* TISTR 2591 improves glucose tolerance in diabetic rats by 32% (Li et al., 2023). These strains act through multi-target pathways: reinforcing intestinal tight junctions to prevent endotoxemia, suppressing pro-inflammatory cytokines (e.g., TNF-*α*, IL-6), and modulating enteroendocrine signaling to enhance insulin secretion (Dong et al., 2023; Zou et al., 2024).

*Akkermansia muciniphila* administration, for example, reduces HbA1c in T2D patients (Liu et al., 2024), while *Bifidobacterium animalis* TISTR 2591 improves glucose tolerance in diabetic rats (Hanchang et al., 2024). Our previous studies have shown that specific drugs can significantly ameliorate disorders of glucose-lipid metabolism induced by daily diet by modulating the lipid metabolism pathway mediated by the gut microbiota (Zhong et al., 2025). These strains act through multi-target pathways: reinforcing intestinal tight junctions to prevent endotoxemia, suppressing pro-inflammatory cytokines (e.g., TNF-*α*, IL-6), and modulating enteroendocrine signaling to enhance insulin secretion. Despite these breakthroughs, microbiota-based therapies remain underutilized in clinical practice due to strain-specific efficacy variability and incomplete mechanistic understanding.

*Lactobacillus*, a genus of Gram-positive lactic acid bacteria, exhibits strong gastrointestinal adaptability and probiotic potential (Chelladhurai et al., 2023). *Lactobacillus helveticus* and *Bifidobacterium longum* have a variety of benefits on human body, including enhancement of human metabolism, improvement of inflammatory response (Ho et al., 2020), regulation of immunity (Xin et al., 2024), hypolipidemic, hypotensive, improvement of carbohydrate and fatty acid metabolism, etc. *L. helveticus* and *Bifidobacterium longum,* when used together, can influence the brain-gut-liver axis, modulate the gut microbiome and the hepatic lipome, along with reduce the neuroinflammation and hippocampal apoptosis (Amaral et al., 2024). *L. helveticus* CD6 reduces total cholesterol (TC), triglyceride (TG), low-density lipoprotein cholesterol (LDL-c) and other biochemical indexes to alleviate high-fat diet-induced hyperlipidaemia (Patil et al., 2021). *In vitro* studies showed that *L. helveticus* can significantly inhibit *α*-amylase and α-glucosidase activity after the addition of oligofructose, showing obvious antidiabetic activity (Kiran et al., 2025). Despite these advances, direct evidence for *Lactobacillus*-mediated antidiabetic effects remains limited, with mechanistic insights into glucose-lipid metabolism pathways incompletely elucidated.

This study investigates the efficacy of *L. helveticus* WIS02, a safe and effective probiotic strain isolated from human breast milk, in streptozotocin (STZ)-induced diabetic mice, focusing on pancreatic islet protection, glycemic control, and lipid regulation. By exploring gut microbiota-mediated mechanisms, we aim to validate the strain’s therapeutic potential and address critical gaps in current probiotic-DM research, ultimately contributing to novel, low-burden interventions for metabolic disorders.

## 2 Materials and methods

### 2.1 Preparation of bacterial suspensions

Glycerol-preserved *L. helveticus* WIS02(CGMCC No. 29243) was provided by MGBlab, Microbiota-Gut-Brain Laboratory, WISBIOM (Bejing) Biotechnology Co. Ltd., and was retrieved from ultra-low temperature storage, thawed at room temperature, and incoculated into deMan-Rogosa-Sharpe (MRS) broth for anaerobic recovery at 37 °C. The strain was subcultured twice with 1% v/v inoculum to ensure viability. Stationary-phase cultures from the second activation were centrifuged at 8,000 × g for 10 min at 4 °C. The viable cell enumeration was determined by the spread plate method, and the plate was incubated for 24 h at 37 °C, and the viable cell number of colonies was counted (CFU/mL). Pellets were resuspended in sterile phosphate-buffered saline (PBS, pH 7.4) to a final concentration of 4 × 10^8^ CFU/mL, and suspensions were freshly prepared for daily animal gavage.

### 2.2 Animals and treatments

The protocol for the experiment received approval from the Experimental Animal Ethics Committee at Tianjin University of Traditional Chinese medicine (approval number: TCM-LAEC2024209F1368), and animal treatment adhered to ethical requirements. Eight-week-old male C57BL/6 mice (26–28 g) were purchased from SPF Biotechnology Co., Ltd. (Beijing, China) and housed in a specific pathogen-free facility at Tianjin University of Traditional Chinese Medicine (12 h light/dark cycle, standard chow diet, sterile water ad libitum).

Following 1 week of acclimatization, mice were randomly assigned to four groups (*n* = 8 per group): normal control group (Norm), diabetes model group (DM), metformin-treated group (Met,200 mg/kg), and *L. helveticus* WIS02 group (WIS02). All interventions were administered daily (0.2 mL/mouse) for 7 consecutive days. On day 10, a single intraperitoneal injection of STZ (150 mg/kg) was used to establish a model in all groups except Norm, which received vehicle control. Fasting blood glucose (FBG) was measured 72 h post-STZ (day 14); mice with FBG > 16.7 mmol/L were considered successfully modeled. Intervention was resumed post-modeling until study endpoint. Daily food/water intake was recorded, with weekly measurements of body weight and FBG. Fresh fecal samples were collected on day 40 and stored at −80 °C. After 12 h fasting, mice were euthanized with 2% pentobarbital sodium (40 mg/kg, i.p.) and blood was collected from the ophthalmic venous plexus for serum separation. Tissues including pancreas, liver, and colon were excised, fixed in 4% paraformaldehyde, or snap-frozen in liquid nitrogen for downstream analyses.

### 2.3 Oral glucose tolerance test

On day 40, mice were fasted for 12 h, followed by oral gavage of glucose solution (2 g/kg body weight). Tail vein blood glucose was measured at 0, 30, 60, and 120 min using a glucometer (Accu-Chek, Roche).

### 2.4 Serum and hepatic biochemical analyses

Serum was separated via centrifugation (7,500 × g, 15 min, 4 °C) and stored at −80 °C. Liver tissues were homogenized in ice-cold PBS, and supernatants were collected post-centrifugation. Commercial kits (Nanjing Jiancheng Bioengineering Institute) were used to quantify serum TC, TG, LDL-c, and insulin, following manufacturer protocols. Hepatic TC, TG, and gluconeogenesis markers were assayed similarly. All measurements were performed in triplicate using a microplate reader (BioTek).

### 2.5 Histopathological examination

Fixed pancreas, liver, and colon tissues were dehydrated in graded ethanol, cleared in xylene, and embedded in paraffin. 5 μm sections were stained with hematoxylin–eosin (H&E) and imaged using a light microscope (Olympus BX53). Pancreatic tissue and liver tissue were photographed at 40X magnification, while intestinal tissue was photographed at 50X magnification.

### 2.6 Shotgun sequencing

The sequencing method for this experiment was analyzed by sequencing with reference to a previously published article (Xu et al., 2023). Fecal microbial DNA was extracted using the QIAamp DNA Stool Mini Kit (QIAGEN) and quantified via NanoDrop 2000 (Thermo Fisher Scientific) and Qubit 3.0 (Thermo Fisher Scientific). Libraries were constructed with the KAPA HyperPlus Library Preparation Kit (KAPA Biosystems) and sequenced on the Illumina NovaSeq 6,000 platform (5 Gb depth per sample) using paired-end 150-bp reads.

The shotgun sequencing was analyzed by Beijing QuantiHealth Technology Co., Ltd. Low-quality reads were removed from the raw data using MOCAT2. Sequencing adapters were removed by Cutadapt software (version v1.14, −m 30), then SolexaQA package was used to remove the reads with a threshold of less than 20 or the length of less than 30 bp. The reads which could be aligned with the mouse genome (*Mus musculus*, GRCm38) were cleaned by using SOAP aligner software (v2.21, −M 4 -l 30 -v 10).

The relative abundance of bacteria was obtained using MetaPhlAn3 software. Based on the taxonomy information, ɑ-diversity was calculated by R package vegan (2.5–6) package and presented by Shannon and Simpson indices. The principal coordinate analysis (PCoA) was calculated based on the Bray-Curtis distance using vegan (2.5–6). Linear discriminant analysis (LDA) Effect Size (LEfSe) method^1^ was used to identify species that show statistically significant differential abundances among groups.

### 2.7 Statistical analysis

Data are presented as mean ± standard deviation (SD). Analyses were performed using SPSS 17.0 and GraphPad Prism 9. Normally distributed data were compared via one-way ANOVA with Newman–Keuls post-hoc test; non-normally distributed data were analyzed via Kruskal-Wallis test. Benjamini-Hochberg FDR correction was applied for Significantly altered strains. Significance was defined as *p* < 0.05 (biochemical data) or adjusted *p* (*q*) < 0.1 (microbial abundance). All final graphs were created using Adobe Illustrator.

## 3 Results

### 3.1 *Lactobacillus helveticus* WIS02 attenuates STZ-induced hyperglycemia and preserves pancreatic

**Figure 1:**
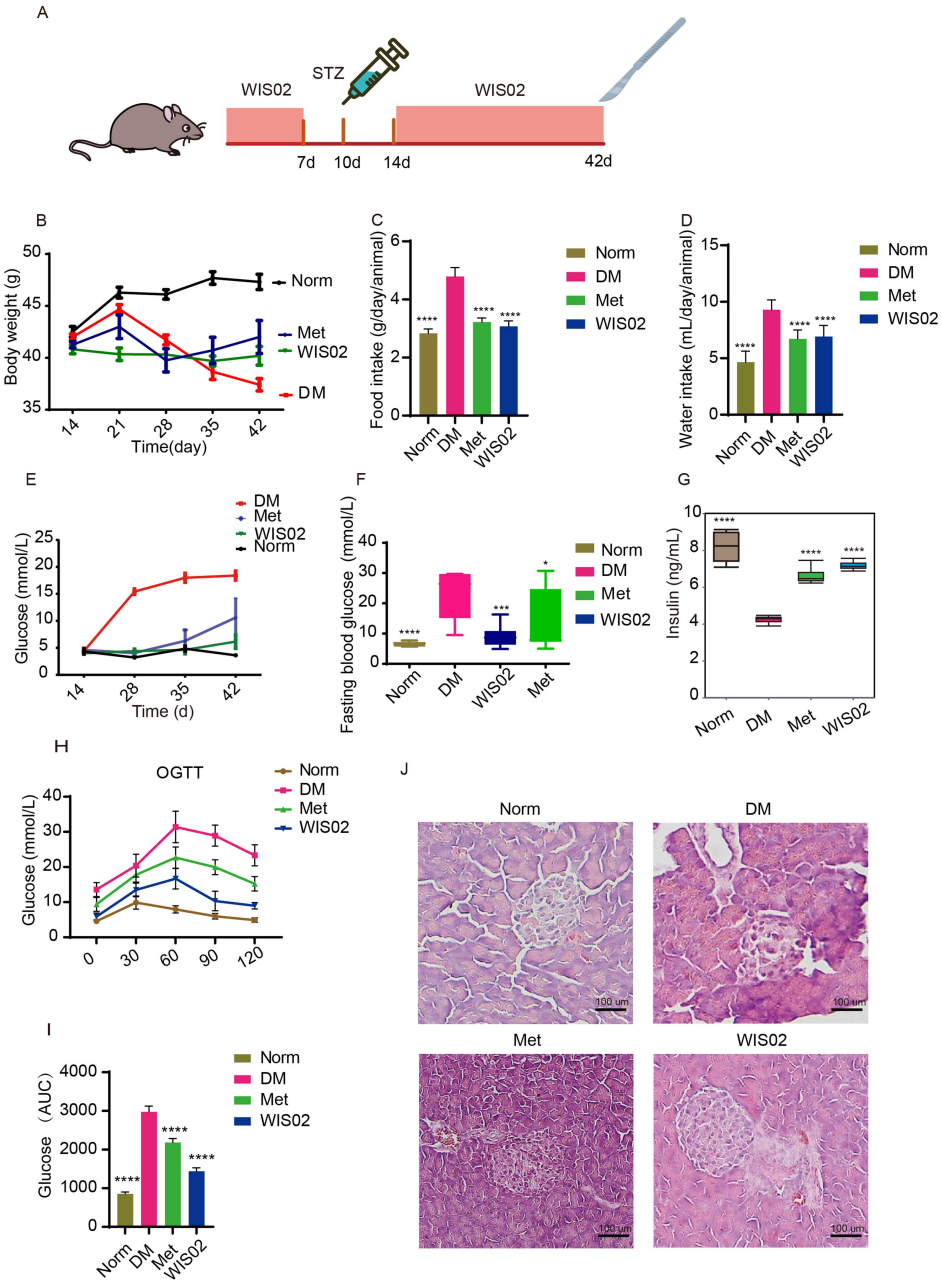
*Lactobacillus helveticus* WIS02 attenuates STZ-induced hyperglycemia and preserves pancreatic function. **(A)** Design of animal experiment. **(B)** Changes in body weight of mice in each group. **(C)** Daily average food intake of mice in each group. **(D)** Daily average water intake of mice in each group. **(E)** Changes in fasting blood glucose (FBG) of mice in each group. **(F)** Fasting blood glucose (FBG) level of mice in each group measured at 42 days. **(G)** Serum insulin level of mice in each group. **(H)** Levels of blood glucose was determined in the different groups using the OGTT. **(I)** AUC of the OGTT was determined. **(J)** Representative pathological changes in pancreas. HE stained sections of the pancreas in magnification 40x. All the data were expressed as mean ± standard deviation (*n* = 10). * *p* < 0.05; ** *p* < 0.01; *** *p* < 0.001;**** *p* < 0.0001.

To evaluate the protective effects of *L. helveticus* WIS02 against pancreatic injury and diabetic progression, a pre-treatment paradigm was employed—mice were administered WIS02 via gavage for 7 days, followed by a 3-day treatment pause prior to intraperitoneal STZ injection (150 mg/kg) to induce *β*-cell damage and hyperglycemia. Fasting blood glucose (FBG) was assessed on day 14 to confirm islet protection, and interventions were resumed for 28 days to evaluate long-term metabolic effects (Figure 1A). Pre-treatment with WIS02 did not alter baseline body weight in non-diabetic mice. Post-STZ induction, the DM group exhibited a transient weight increase followed by rapid loss, accompanied by marked hyperphagia and polydipsia. In contrast, WIS02-treated mice maintained stable weight gain throughout the study and displayed significantly reduced food/water intake compared to the DM group, approaching levels observed in Norm mice (Figures 1B–D). STZ injection triggered a sharp rise in FBG in the DM group, peaking at 18.3 mmol/L by day 42. Notably, WIS02 pre-treatment blunted this hyperglycemic response, that is, FBG levels in the WIS02 group were 66.0% lower than the DM group at day 42 (5.14 ± 2.15 mmol/L), even outperforming the Met group by day 42 (Figures 1E,F). Correspondingly, serum insulin levels in the DM group plummeted to 69.2% of Norm values, indicating severe *β*-cell dysfunction. In contrast, WIS02 and metformin both preserved insulin secretion, suggesting superior islet protection (Figure 1G). Simultaneously, OGTT assay demonstrated that the oral glucose tolerance of the animals could be significantly improved by WIS02 and metformin (Figures 1H,I). The pancreatic islets of normal group mice were structurally intact with clear edges, round or oval shapes were visible, the cells were arranged in a regular manner. Mice in the DM group showed severe structural destruction of the islets, islet atrophy, irregular arrangement of islet cells, and the presence of cellular edema compared to the pancreas of normal mice. The *L. helveticus* WIS02 treatment had a significant protective effect on the pancreatic islets. The pancreas of mice showed obvious islet structure, clearer islet edges, and lower cell edema in WIS02 group. It significantly alleviated the islet tissue damage affected by STZ and largely protected the islet tissue structure, enabling the islets to maintain their basic function (Figure 1J). These results suggest that *L. helveticus* WIS02 significantly protects the pancreas from STZ, prevents the increase of blood glucose, and it improves the body weight and diet of animals, demonstrating excellent efficacy in managing hyperglycemia.

### 3.2 *Lactobacillus helveticus* WIS02 improves dyslipidemia in diabetic mice

**Figure 2:**
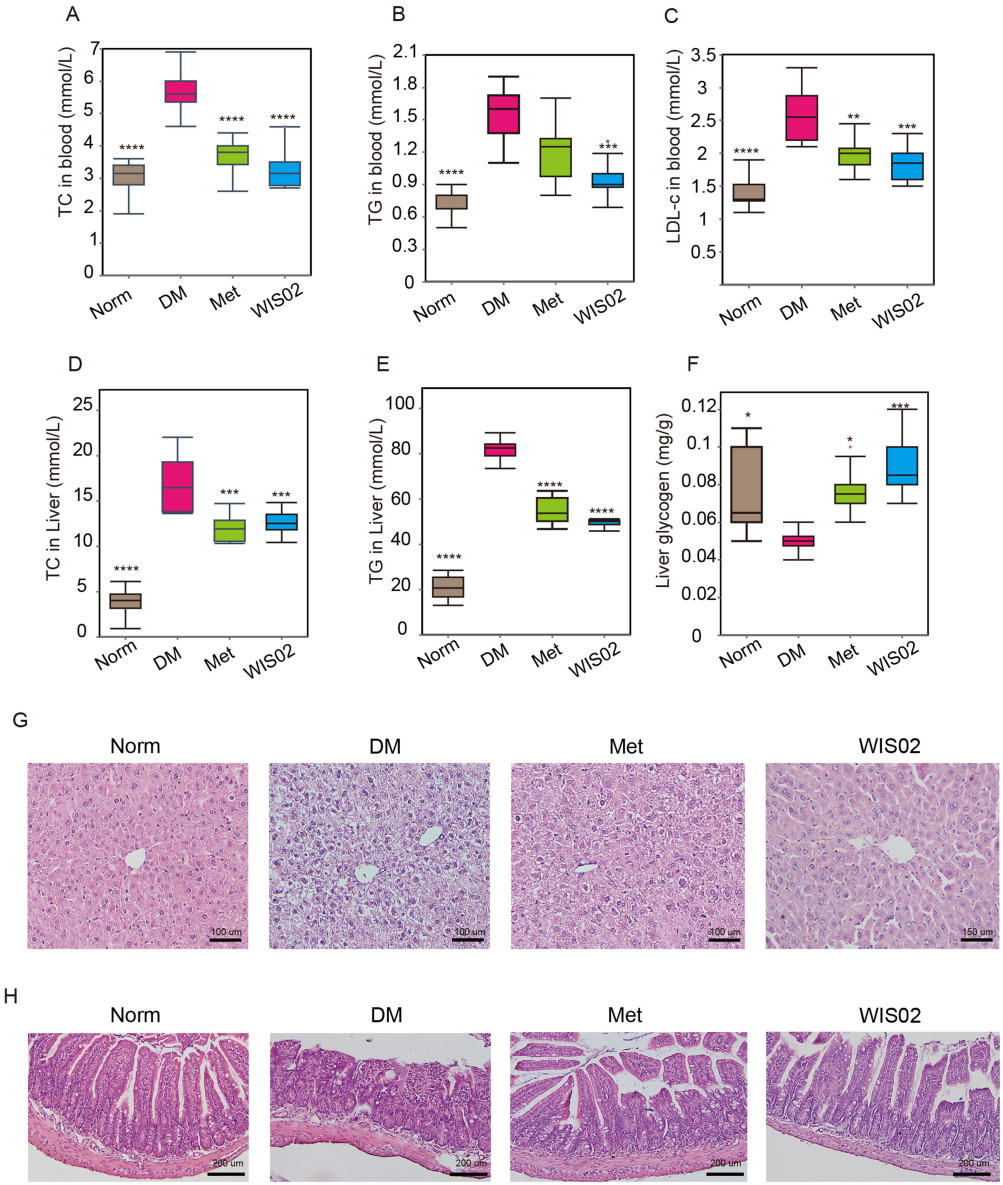
*Lactobacillus helveticus* WIS02 improves dyslipidemia in diabetic mice. **(A)** Changes in serum TC levels in each group of mice. **(B)** Changes in serum TG levels in each group of mice. **(C)** Changes in serum LDL-c levels in each group of mice. **(D)** Changes in TC levels in the livers of mice in each group. **(E)** Changes in TG levels in the livers of mice in each group. **(F)** Changes in liver glycogen levels in the livers of mice in each group. **(G)** Representative pathological changes in the liver. Liver HE-stained sections were observed at 40 × magnification. **(H)** Representative pathological changes in the intestine. HE-stained intestinal sections were observed at 50 × magnification. All data are expressed as mean ± standard deviation (*n* = 10). * *p* < 0.05; ** *p* < 0.01; *** *p* < 0.001;**** *p* < 0.0001.

Dyslipidemia, a key comorbidity of diabetes, accelerates vascular complications and disease progression. We therefore investigated whether WIS02 modulates lipid metabolism in STZ-induced diabetic mice. STZ induction significantly elevated serum TC, TG, and LDLC in the DM group compared to Norm mice (*p* < 0.001 for all) (Figures 2A–C). While metformin treatment reduced serum TC and LDL-c (*p* < 0.05 vs. DM), it failed to normalize TG levels (Figures 2A–C). In contrast, WIS02 administration led to a comprehensive reduction in serum lipids, including TC (−23.6%), TG (−39.5%), and LDL-C (−28.0%) compared to the DM group (*p* < 0.01 for all), with TC levels approaching Norm values (Figures 2A–C). Meanwhile, the liver TC and TG levels of diabetic animals were also significantly higher than those of normal mice, and *L. helveticus* WIS02 treatment significantly reduced the liver TC and TG levels with better effects than metformin (Figures 2D,E). In addition, hepatic glycogen content was significantly reduced in diabetic mice, and *L. helveticus* WIS02 significantly increased hepatic glycogen content to levels close to those of normal mice (Figure 2F). Furthermore, DM mice showed extensive hepatic damage, including disorganized hepatocytes, nuclear fragmentation, cytoplasmic vacuolation (indicative of lipid droplet accumulation), and interstitial edema. WIS02 intervention restored hepatic architecture: hepatocytes regained polygonal morphology, nuclei appeared intact, and vacuolation was reduced compared to the DM group. These improvements align with the observed reductions in hepatic TC and TG, confirming mitigation of lipid-induced liver injury (Figure 2G). At the same time, diabetes also led to the destruction of the intestinal structure in mice. Compared with normal mice, the colonic tissue structure of mice in the DM group was disrupted, with structural incompleteness, irregular cellular arrangement, a large number of inflammatory cells infiltration, and severe destruction of crypt structure. Gavage of *L. helveticus* WIS02 resulted in mice with intact colon tissues, clear and well-arranged villus structure, and less destruction of crypt structure. This suggests that *L. helveticus* WIS02 may be able to maintain the integrity of the intestinal tissues and improve the ability of lower than diabetes mellitus (Figure 2H). These results suggest that *L. helveticus* WIS02 significantly ameliorates dyslipidemia in diabetic mice and mitigates tissue damage caused by hyperglycemia.

### 3.3 *Lactobacillus helveticus* WIS02 tends to favor the gut flora of diabetic animals

**Figure 3:**
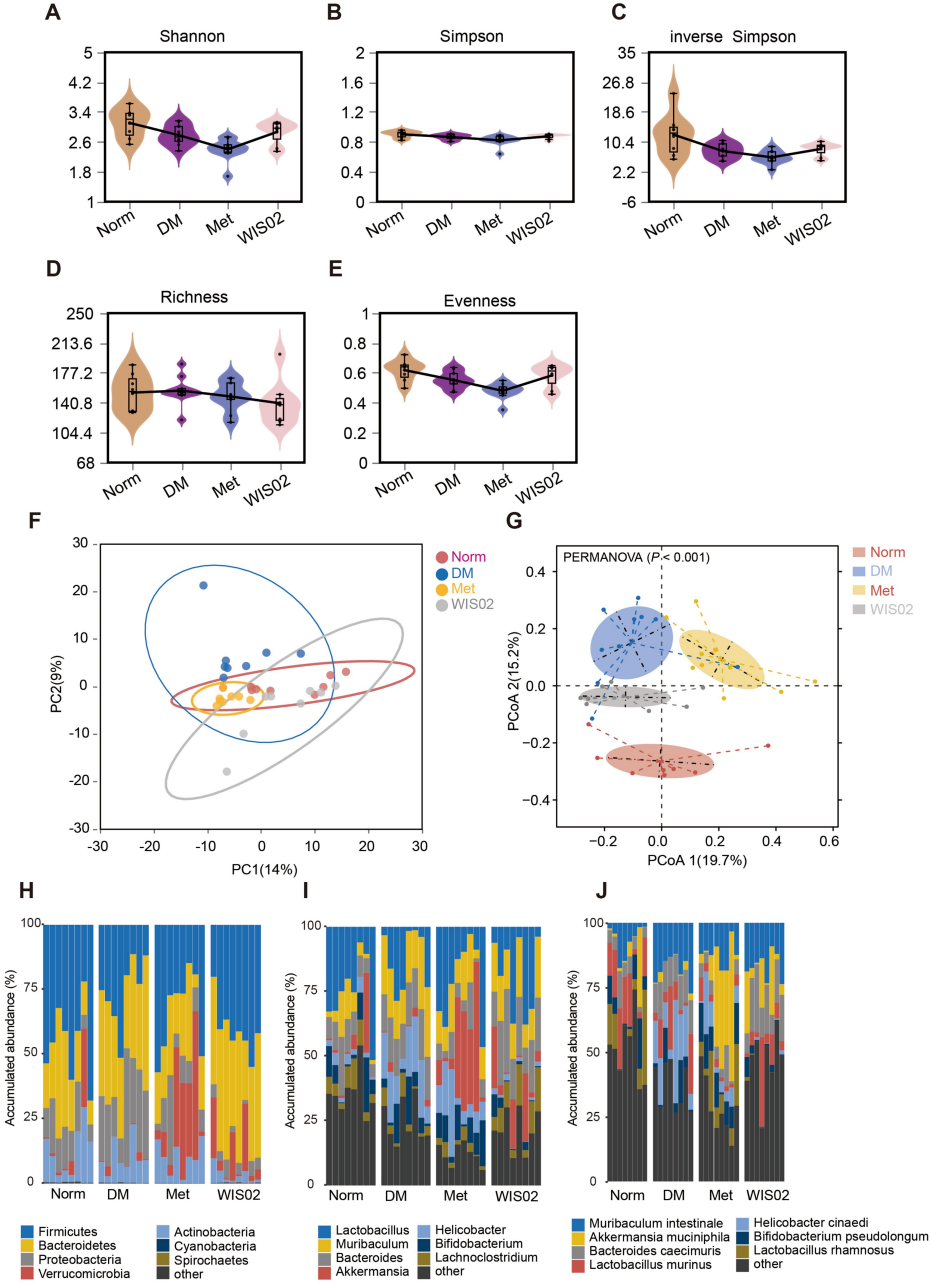
*Lactobacillus helveticus* WIS02 regulates intestinal flora diversity. **(A)** Shannon curves. **(B)** Simpson analysis. **(C)** Inverse Simpson analysis. **(D)** Richness analysis. **(E)** Evenness analysis. **(F)** PCA analysis. **(G)** PCoA analysis. **(H)** Changes in the abundance of gut microbiota at the phylum level in each group of mice. **(I)** Changes in the abundance of gut microbiota at the genus level in each group of mice. **(J)** Changes in the abundance of gut microbiota at the species level in each group of mice. All the data were expressed as mean ± standard deviation (*n* = 10). * *p* < 0.05; ** *p* < 0.01; *** *p* < 0.001.

There is a close relationship between gut microorganisms and the development of diabetes. Analysis of gut flora showed that diabetic mice had a lower diversity of flora (Shannon index, Simpson index, Evenness index) than the normal group. Metformin treatment further reduced the diversity of the flora, whereas *L. helveticus* WIS02 treatment had less effect on alpha diversity (Figures 3A–E). Although WIS02 had a small effect on the alpha diversity of the flora, it significantly altered the overall structure of the flora. Principal coordinate analysis showed that the DM mice colony was significantly separated from the normal mouse colony, and metformin treatment resulted in lateral separation of the colony from the model group, while WIS02 treatment shifted the overall colony structure toward the normal group (Figures 3F,G).

We further compared the differences in the composition of the gut microbiota of the different groups at the level of phylum, genus and species. At the phylum level, the DM group showed elevated levels of *Proteobacteria* and reduced levels of *Firmicutes* and *Actinobacteria* compared to the normal group. Metformin treatment significantly reduced the abundance of *Proteobacteria* while substantially elevating the abundance of *Verrucomicrobia*. In contrast, *L. helveticus* WIS02 treatment further reduced the abundance of *Proteobacteria* while increasing the level of *Bacteroidetes* and it also increased the level of *Verrucomicrobia* (Figure 3H).

At the genus and species level (Figures 3I,J), the DM group mainly increased the abundance of *Lactobacillus*, *Muribaculum* and *Helicobacter*, especially *Muribaculum Intestinale* and *Helicobacter cinaedi*, while *Akkermansia muciniphila* was significantly reduced in abundance. Metformin treatment substantially increased the abundance of *Akkermansia muciniphila*, consistent with previous reports. But at the same time, the abundance of *Helicobacter*, especially *Helicobacter cinaedi*, was also high in the metformin group, which was close to the model group. On the contrary, *L. helveticus* WIS02 treatment significantly decreased the abundance of *Helicobacter cinaedi* and increased the abundance of *Bacteroidetes*, especially *Bacteroides caecimuris*. Also, WIS02 treatment increased the abundance of *Akkermansia muciniphila*. These results indicate that *L. helveticus* WIS02 significantly promotes the growth of beneficial bacteria such as *Bacteroides* and *Akkermansia* and it reduces the abundance of harmful bacteria *Proteobacteria*, especially *Helicobacter*, and maintains the homeostasis of the intestinal microenvironment.

**Figure 4:**
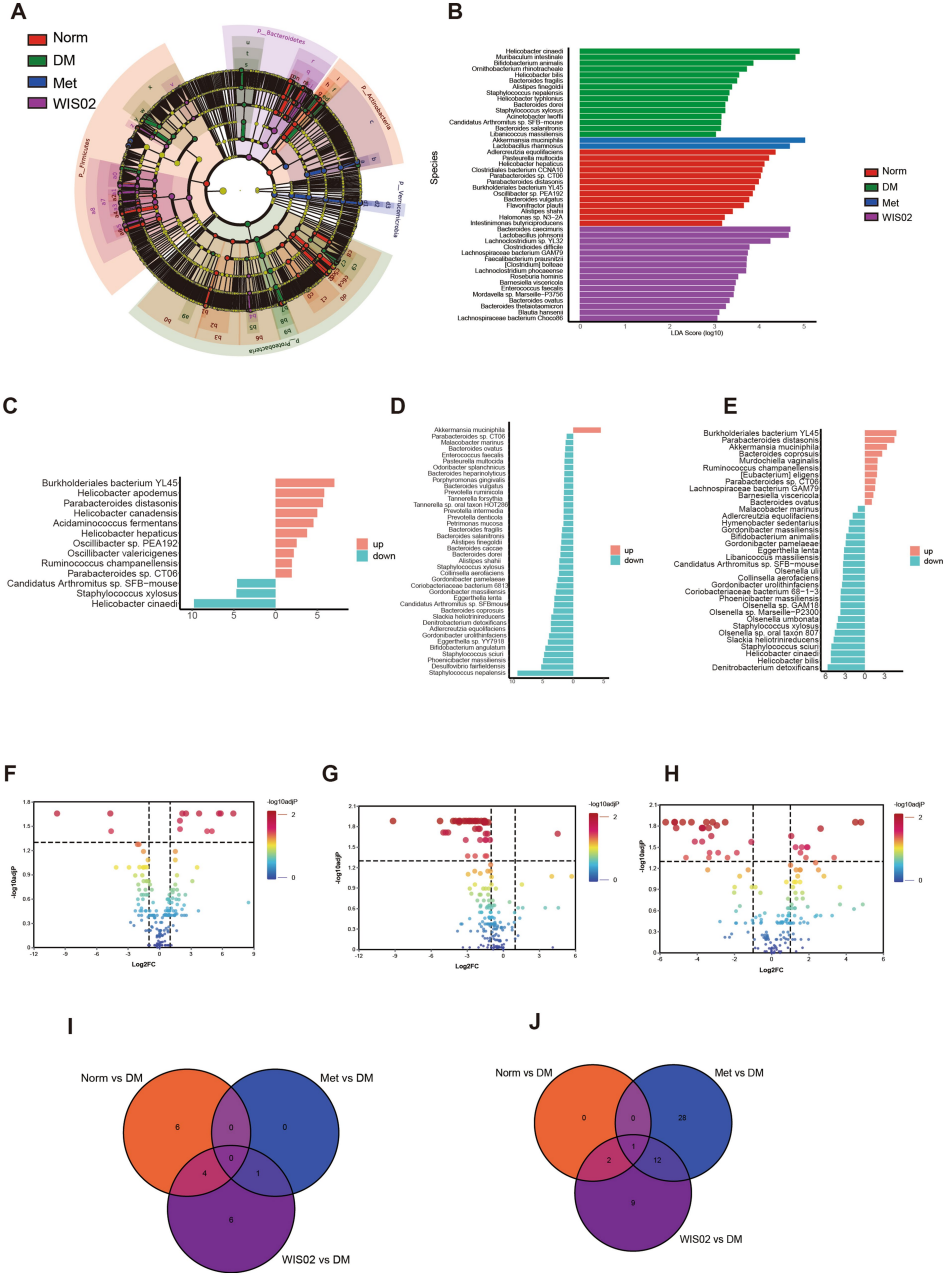
*Lactobacillus helveticus* WIS02 tends to favor the gut flora of diabetic animals. **(A)** LEfSe analysis. **(B)** LDA analyses. **(C)** Significant changes in the microbiota between the Norm group and the DM group. **(D)** Significant changes in the microbiota between the Met group and the DM group. **(E)** Significant changes in the microbiota between the WIS02 group and the DM group. **(F)** Changes in the intestinal microbiota between the Norm group and the DM group. **(G)** Changes in the intestinal microbiota between the Met group and the DM group. **(H)** Changes in the intestinal microbiota between the WIS02 group and the DM group. **(I)** VEEN analysis of upward adjustments in microbial communities between groups. **(J)** VEEN analysis of downregulation in microbial communities between groups. All the data were expressed as mean ± standard deviation (*n* = 10). * *p* < 0.05; ** *p* < 0.01; *** *p* < 0.001.

Linear discriminant analysis Effect Size (LEfSe) and Linear Discriminant Analysis of Effect Size (LDA) analyses further differentiated the main regulators of WIS02. The DM group was mainly enriched *with Helicobacter canadensis*, *Muribaculum intestinale*, *Staphylococcus* and other opportunistic pathogenic bacteria. Metformin group was mainly enriched with beneficial bacteria such as *Akkermansia muciniphila*, *Lactobacillus rhamnosus*, While *L. helveticus* WIS02 group was mainly enriched with *Bacteroides caecimuris*, *Lactobacillus jensenii*, *Lachnoclostridium*, *Faecalibacterium prausnitzii*, *Lachnoclostridium phocaeense* and other short-chain fatty acid-producing bacteria (Figures 4A,B). Comparison of the normal and administered groups with the diabetes model control group, respectively, showed that the abundance of beneficial bacteria such as *Parabacteroides distasonis* and *Oscillibacter* spp. was significantly higher in the normal group than in the diabetes model group. While the two very low abundance Helicobacter strains were also enriched, but the main Helicobacter species *Helicobacter canadensis*, on the other hand, was heavily enriched in DM (Figures 4C,F). Compared to the DM group, the metformin group was significantly enriched in only one bacterium, namely *Akkermansia muciniphila* (Figures 4D,G). In contrast, the WIS02 group was not only enriched *with Akkermansia muciniphila*, but also significantly enriched with abundant bacteria such as *Parabacteroides distasonis* and Oscillibacter spp. in the normal group (Figures 4E,H). These results suggest that WIS02 not only significantly restores the gut microbiota of diabetic mice to normal levels, but also further increases the abundance of beneficial bacteria such as *Akkermansia muciniphi*, *Bacteroides ovatus* and *Bacteroides coprosuis*, thereby positively regulating glucose and lipid metabolism (Figure 4E).

### 3.4 *Lactobacillus helveticus* WIS02 improves DM-related biochemical indices by regulating gut microbiota

**Figure 5:**
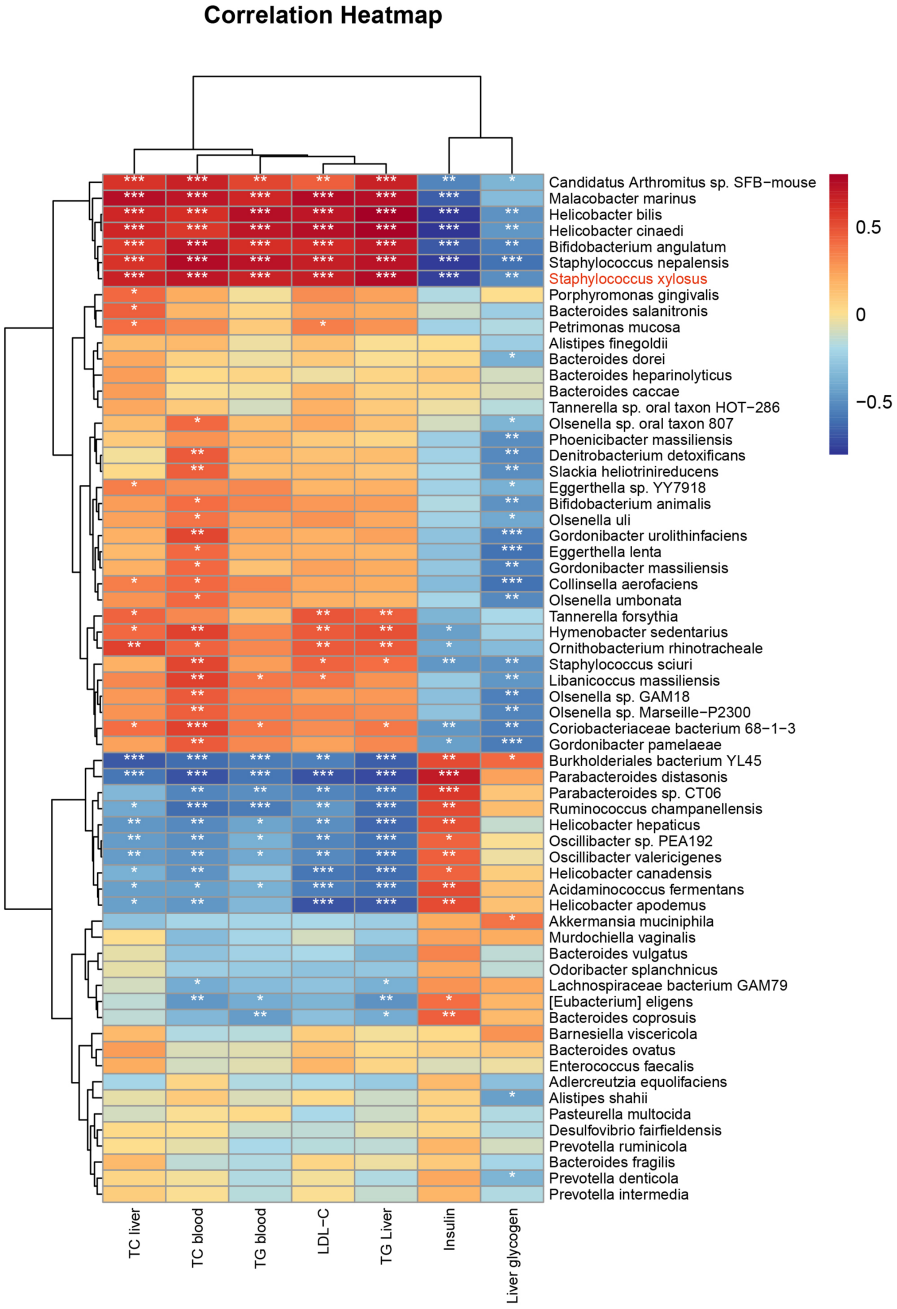
Correlation heatmap displaying relationships between various bacteria species and health indicators such as TC liver, TC blood, and others. Colors range from red (strong positive correlation) to blue (strong negative correlation). * *p* < 0.05; ** *p* < 0.01; *** *p* < 0.001; **** *p* < 0.0001.

To further explore the relationship between WIS02 regulation of gut microbiota and its alleviation of diabetes, we performed Spearman’s correlation analysis of specific differences in strains with physiological indicators of diabetes (Figure 5). Serum and liver levels of TC, TG, and serum LDL-C expression were all correlated with *Candidatus Arthromitus* sp. *SFB-mouse*, *Malacobacter marinus*, *Helicobacter bilis*, *Helicobacter cinaedi*, *Bifidobacterium angulatum*, *Staphylococcus nepalensis*, *Staphylococcus xylosus* expression levels were positively correlated with *Burkholderiales bacterium YL45*. *Parabacteroides distasonis* expression levels were negatively correlated. The insulin levels were positively correlated with the expression levels of *Candidatus Arthromitus* sp. *SFB-mouse*, *Malacobacter marinus*, *Helicobacter bilis*, *Helicobacter cinaedi*, *Bifidobacterium angulatum*, *Staphylococcus nepalensis*, *Staphylococcus xylosus* expression levels were negatively correlated with those of *Burkholderiales bacterium YL45*, *Parabacteroides distasonis*, *Parabacteroides* sp. *Parabacteroides* sp. *CT06* were positively correlated. Therefore, increasing the levels of *Burkholderiales bacterium YL45*, *Parabacteroides distasonis*, *Parabacteroides* sp. *CT06* and decreasing the levels of *Candidatus Arthromitus* sp. *SFB-mouse*, *Malacobacter marinus*, *Helicobacter* spp., *Staphylococcus* spp. contributed to the increase of insulin levels and decrease of lipid levels.

## 4 Discussion

This study systematically investigates the therapeutic potential of *L. helveticus* WIS02 in STZ-induced diabetic mice, employing a pre-treatment and long-term intervention paradigm to evaluate its multi-faceted effects on metabolic homeostasis, tissue protection, and gut microbiota modulation. Our findings demonstrate that WIS02 not only ameliorates hyperglycemia and dyslipidemia but also preserves pancreatic, hepatic, and intestinal structural integrity while reshaping the gut microbial ecosystem—collectively highlighting its potential as a novel probiotic intervention for diabetes.

*Lactobacillus helveticus* is a Gram-positive bacterium in the genus *Lactobacillus*, which is widely found in fermented dairy products and mammalian intestines. *L. helveticus* is known for its acid and bile salt resistance and strong protein hydrolysis activity, capable of breaking down milk proteins to release biologically active peptides. It is often used in food and probiotics. It has been found that *L. helveticus* LZ-R-5 can produce bioactive extracellular polysaccharides, which have anti-inflammatory and antibacterial bioactivities, regulate the immune function, and alleviate the inflammatory response of the body (Zhao et al., 2025). With the further research direction, *L. helveticus* showed significant hypoglycemic and hypolipidemic activities, which can alleviate the tissue damage induced by high glucose and metabolic disorders induced by high glucose (Korkmaz et al., 2019). Studies have shown that the intake of *L. helveticus* CD6 can significantly reduce the blood lipid level of mice on a high-fat diet, reduce the change in body weight of the animals, adjust the metabolism level and reduce the liver damage (Patil et al., 2021), and the anti-lipotropic and lipid metabolism regulating ability of *Lactobacillus suis* HY7804 is superior to that of the common *Lactobacillus plantarum* LP158 and *Lactobacillus paracasei* LPC226 in comparison. Long-term intake of *L. helveticus* is necessary for the study of *Lactobacillus* as a functional probiotic. Studies have shown that long-term intake of *L. helveticus* not only regulated the body’s glucose-lipid metabolism and adjusted the body’s gut microbiota, but also enriched the bioavailability of DHA and omega-3 fatty acids in the retina of mice, which helped to maintain the health of the body (Lapaquette et al., 2024). In our study, we found that *L. helveticus* WIS02 also possessed similar pharmacological activities, and supplementation with WIS02 significantly lowered blood glucose levels, significantly reduced the levels of TC, TG and other physiological indicators in serum and liver, and effectively alleviated the disorders of glucose and lipid metabolism in mice.

Numerous studies have found that the increasing prevalence of diabetes mellitus and the average age of patients tends to be younger (Hoyek et al., 2024), which has become the focus of research in recent years. Recent macro-genomics studies have revealed significant differences in the gut microbiota between healthy people and diabetic patients, suggesting that there is a significant association between the development of diabetes mellitus and the ecological dysregulation of gut microbiota. Probiotics have become an important biological tool for regulating flora-host interactions due to their unique intestinal luminal colonization advantages, and probiotics have the qualities of stability and efficacy in the gastrointestinal environment compared to drugs, and do not have the adverse effects produced by drug interventions, so probiotics have become a new approach to prevent diabetes (Qin et al., 2025). Recent studies have shown that a variety of probiotics exhibit significant hypoglycemic effects in diabetic mice and have a positive impact on the treatment of diabetes (Li et al., 2024). *Lactobacillus casei* Q14 was found to have the potential to alleviate diabetes in rats by improving blood glucose and pancreatic islet levels, affecting the expression of genes involved in hepatic gluconeogenesis-related genes, and promoting the remodeling of gut microbiota structure (Qu et al., 2018). In this study, we found that the abundance of *Lactobacillus* species also exhibited significant changes influenced by WIS02. We hypothesize that this may accelerate the conversion of conjugated bile acids to free bile acids within the gut. This process could potentially improve STZ-induced diabetes by enhancing bile acid signaling, modulating metabolic regulation synergistically, and influencing enzyme activity in gut microbiota (Gadaleta et al., 2022; Crudele et al., 2023). *Akkermansia muciniphila*, a probiotic that is often used as a research target, was found to have a significant ameliorative effect on a variety of metabolic disorders in experiments, with a particularly prominent effect on glucose and lipid metabolism. The STZ-induced mouse hyperglycemia model is a commonly used modeling approach in research. It specifically targets and destroys insulin-secreting *β* cells in the pancreas, leading to decreased insulin levels in the body and inducing persistent, insulin-dependent hyperglycemia. After 5 months of sustained high-glucose status in STZ-modeled mice, there was a significant increase in the abundance of *Akkermansia muciniphila* in the intestines of the mice, as well as an increased production of short-chain fatty acids *in vivo* (Zhang et al., 2024), which also suggests that *Akkermansia muciniphila* abundance in vivo is closely related to the development of diabetes. In addition, *Akkermansia muciniphila* has the effects of repairing the intestinal barrier, reducing metabolically induced inflammation, as well as improving pancreatic β-cell apoptosis and increasing pancreatic islet cell secretion in high-fat diet-induced prediabetic mice, which can effectively delay and reverse the progression of prediabetes (Yan et al., 2024). This phenomenon was also observed in this study.

Enrichment for flora changes revealed that *Staphylococcus xylosus* was the strain that was significantly down-regulated in all groups. *Staphylococcus xylosus* is a common *staphylococcus* with properties that is resistant to salt and heat (Battaglia and Garrett-Sinha, 2023) at wounds and to promote inflammation and progression (Li et al., 2022) in medical studies. It has been suggested that high fat may cause *Staphylococcus xylosus* strain abundance levels in vivo, and that reduced *Staphylococcus xylosus* abundance may be associated with inhibition of fat accumulation (Guo et al., 2023). A common phenotype in diabetes is slow wound healing, which triggers ulcers, such as diabetic foot ulcers, and in related studies, *Staphylococcus xylosus* was found to colonize wounds, delaying barrier repair and prolonging inflammatory healing (Khadka et al., 2024), whereas high oxidative stress is able to have an impact on the organism’s microbiota, decreasing the bacterial flora that may influence the healing process (Kim et al., 2020). In this study, it was found that *L. helveticus* WIS02 can promote oxidative stress in the body, exerting anti-inflammatory and antioxidant effects. The abundance of *Staphylococcus xylosus* was significantly reduced, which prevents inflammatory responses in the body and potentially lowers the risk of diabetes complications. We found that *Staphylococcus xylosus* was analyzed by biochemical level enrichment, which showed a positive correlation with TC, TG and LDL-C, and a negative correlation with insulin and hepatic glycogen expression. This suggests that changes in abundance of *Staphylococcus xylosus* are closely related to glycolipid metabolism and it may be a key group of bacteria for assessing glycolipid metabolism.

Despite the compelling metabolic and histological improvements observed with *L. helveticus* WIS02, this study has three key limitations that warrant consideration and guide future inquiry. Firstly, while WIS02 robustly ameliorates hyperglycemia and dyslipidemia, the molecular pathways underlying these effects remain incompletely characterized. Notably, we did not validate downstream effectors of glucose homeostasis (e.g., AMPK/AKT signaling, GLUT4 translocation) or lipid metabolism (e.g., PPARα/*γ* activation, SREBP-1c suppression) in hepatic or adipose tissues. Without such mechanistic interrogation, it is challenging to distinguish whether WIS02 acts directly on host metabolic machinery or indirectly via microbial metabolites (e.g., short-chain fatty acids, indole derivatives). Secondly, our 16S rRNA sequencing data demonstrate that WIS02 reshapes the gut microbiota, enriching beneficial taxa (e.g., *Akkermansia*, *Lactobacillus*) and depleting pro-inflammatory pathobionts (e.g., *Escherichia coli*). However, we did not perform functional validation to confirm whether these microbial shifts are mechanistically linked to WIS02’s metabolic benefits. We regret that we did not conduct research or validation on the relevant mechanisms. In subsequent studies, we will incorporate related content to explore their mechanisms of action and enhance the completeness of our experiments. Critical experiments—such as fecal microbiota transplantation (FMT) from WIS02-treated mice to germ-free recipients, or targeted depletion of key taxa using antibiotics—are needed to establish causality. Additionally, metagenomic and metatranscriptomic analyses could identify microbial enzymes that contribute to WIS02’s efficacy, bridging gaps between microbial composition and host phenotype. This will also be the main focus of our future research. We established a hyperglycemic model in mice using streptozotocin (STZ). An induction dose of 150 mg/kg was selected for this experiment, taking into account both the toxic effects of STZ on organs such as the liver and the mortality risk to mice due to blood glucose fluctuations. Thus, a relatively high yet safe dose was chosen to establish the hyperglycemic model under equivalent experimental conditions. However, the single-dose high-dose STZ model employed in this study primarily simulates a state of absolute insulin deficiency characterized by rapid and extensive destruction of *β*-cells. Its pathophysiology more closely resembles that of type 1 diabetes or the advanced stage of type 2 diabetes with severe β-cell failure. Furthermore, rodent models exhibit interspecies differences in gut microbial composition, nutrient metabolism, and immune responses compared to humans, limiting direct extrapolation of WIS02’s efficacy. To address this, future work should include: (1) studies in diet-induced obese (DIO) mice or Zucker diabetic fatty (ZDF) rats to model of diabetes; (2) dose-escalation studies to determine optimal therapeutic windows; and (3) ultimately, randomized controlled trials (RCTs) in prediabetic, with stratification by baseline microbiota profiles to identify responders.

## 5 Conclusion

*Lactobacillus helveticus* WIS02 ameliorates STZ-induced hyperglycemia, dyslipidemia, and tissue damage in mice by regulating glycolipid metabolism, protecting pancreatic/hepatic/intestinal integrity, and remodeling gut microbiota. Despite limitations in mechanistic validation, microbiota causality, and clinical translation, this strain shows promise as a multi-targeted probiotic for metabolic disorders.

## Data Availability

Upon reasonable request, the data can be obtained from the corresponding author.

## Footnotes

1
https://github.com/biobakery/lefse

## References

[ref1] Amaral W. Z. Kokroko N. Treangen T. J. Villapol S. Gomez-Pinilla F. (2024). Probiotic therapy modulates the brain-gut-liver microbiota axis in a mouse model of traumatic brain injury. Biochim. Biophys. Acta (BBA) - Mol. Basis Dis. 1870:167483. doi: 10.1016/j.bbadis.2024.167483, 39209236 PMC11526848

[ref2] Bailey C. J. (2024). Metformin: therapeutic profile in the treatment of type 2 diabetes. Diabetes Obes. Metab. 26, 3–19. doi: 10.1111/dom.15663, 38784991

[ref3] Bailey T. S. Mezitis N. H. (1990). Combination therapy with insulin and sulfonylureas for type II diabetes. Diabetes Care 13, 687–695. doi: 10.2337/diacare.13.6.687 2192852

[ref4] Battaglia M. Garrett-Sinha L. A. (2023). Staphylococcus xylosus and *Staphylococcus aureus* as commensals and pathogens on murine skin. Lab. Anim. Res. 39:18. doi: 10.1186/s42826-023-00169-0, 37533118 PMC10394794

[ref5] Chelladhurai K. Ayyash M. Turner M. S. Kamal-Eldin A. (2023). *Lactobacillus helveticus*: health effects, current applications, and future trends in dairy fermentation. Trends Food Sci. Technol. 136, 159–168. doi: 10.1016/j.tifs.2023.04.013

[ref6] Chen K. Wang H. Yang X. Tang C. Hu G. Gao Z. (2024). Targeting gut microbiota as a therapeutic target in T2DM: A review of multi-target interactions of probiotics, prebiotics, postbiotics, and synbiotics with the intestinal barrier. Pharmacol. Res. 210:107483. doi: 10.1016/j.phrs.2024.107483, 39521027

[ref7] Crudele L. Gadaleta R. M. Cariello M. Moschetta A. (2023). Gut microbiota in the pathogenesis and therapeutic approaches of diabetes. EBioMedicine 97:104821. doi: 10.1016/j.ebiom.2023.104821, 37804567 PMC10570704

[ref8] Dong C. Yang Y. Wang Y. Hu X. Wang Q. Gao F. . (2023). Gut microbiota combined with metabolites reveals unique features of acute myocardial infarction patients different from stable coronary artery disease. J. Adv. Res. 46, 101–112. doi: 10.1016/j.jare.2022.06.008, 35750287 PMC10105070

[ref9] Duan Y. Wu X. Yang Y. Gu L. Liu L. Yang Y. . (2022). Marked shifts in gut microbial structure and neurotransmitter metabolism in fresh inmates revealed a close link between gut microbiota and mental health: A case-controlled study. Int. J. Clin. Health Psychol. 22:100323. doi: 10.1016/j.ijchp.2022.100323, 35892042 PMC9289638

[ref10] Gadaleta R. M. Cariello M. Crudele L. Moschetta A. (2022). Bile salt hydrolase-competent probiotics in the management of IBD: unlocking the "bile acid code". Nutrients 14:3212. doi: 10.3390/nu14153212, 35956388 PMC9370712

[ref11] Gómez-Pérez A. M. Muñoz-Garach A. Lasserrot-Cuadrado A. Moreno-Indias I. Tinahones F. J. (2024). Microbiota transplantation in individuals with type 2 diabetes and a high degree of insulin resistance. Nutrients 16:3491. doi: 10.3390/nu16203491, 39458486 PMC11510444

[ref12] Guo Y. Liu M. Liu X. Zheng M. Xu X. Liu X. . (2023). Metagenomic and untargeted metabolomic analysis of the effect of Sporisorium reilianum polysaccharide on improving obesity. Foods 12:1578. doi: 10.3390/foods12081578, 37107373 PMC10137368

[ref13] Hanchang W. Dissook S. Wongmanee N. Rojanaverawong W. Charoenphon N. Pakaew K. . (2024). Antidiabetic effect of *Bifidobacterium animalis* TISTR 2591 in a rat model of type 2 diabetes. Probiotics Antimicrob. Proteins 17, 4298–4313. doi: 10.1007/s12602-024-10377-2 39384734

[ref14] Ho S. W. El-Nezami H. Shah N. P. (2020). The protective effects of enriched citrulline fermented milk with *Lactobacillus helveticus* on the intestinal epithelium integrity against *Escherichia coli* infection. Sci. Rep. 10:499. doi: 10.1038/s41598-020-57478-w, 31949265 PMC6965087

[ref15] Hossain M. J. Al-Mamun M. Islam M. R. (2024). Diabetes mellitus, the fastest growing global public health concern: early detection should be focused. Health Sci. Rep. 7:e2004. doi: 10.1002/hsr2.2004, 38524769 PMC10958528

[ref16] Hoyek K. Libman I. Mkparu N. Hong Y. H. Arslanian S. Vajravelu M. E. (2024). Child opportunity index and clinical characteristics at diabetes diagnosis in youth: type 1 diabetes versus type 2 diabetes. BMJ Open Diabetes Res. Care 12:e003968. doi: 10.1136/bmjdrc-2023-003968, 38631820 PMC11029253

[ref17] Iatcu O. C. Hamamah S. Covasa M. (2024). Harnessing prebiotics to improve type 2 diabetes outcomes. Nutrients 16:3447. doi: 10.3390/nu16203447, 39458444 PMC11510484

[ref18] Khadka V. D. Markey L. Boucher M. Lieberman T. D. (2024). Commensal skin bacteria exacerbate inflammation and delay skin barrier repair. J. Invest. Dermatol. 144, 2541–2552.e10. doi: 10.1016/j.jid.2024.03.033, 38604402

[ref19] Kim J. H. Ruegger P. R. Lebig E. G. VanSchalkwyk S. Jeske D. R. Hsiao A. . (2020). High levels of oxidative stress create a microenvironment that significantly decreases the diversity of the microbiota in diabetic chronic wounds and promotes biofilm formation. Front. Cell. Infect. Microbiol. 10:259. doi: 10.3389/fcimb.2020.00259, 32582564 PMC7283391

[ref20] Kiran S. Sreeja V. Patel H. K. (2025). In vitro probiotic and bio-functional properties of a synbiotic composed of *Lactobacillus helveticus* MTCC 5463 and Fructo-oligosaccharide. Food Biosci. 63:105747. doi: 10.1016/j.fbio.2024.105747

[ref21] Korkmaz O. A. Sumlu E. Koca H. B. Pektas M. B. Kocabas A. Sadi G. . (2019). Effects of Lactobacillus plantarum and *Lactobacillus helveticus* on renal insulin signaling, inflammatory markers, and glucose transporters in high-fructose-fed rats. Medicina 55:207. doi: 10.3390/medicina55050207, 31137715 PMC6572085

[ref22] Lapaquette P. Terrat S. Proukhnitzky L. Martine L. Grégoire S. Buteau B. . (2024). Long-term intake of *Lactobacillus helveticus* enhances bioavailability of omega-3 fatty acids in the mouse retina. NPJ Biofilms Microbiomes 10:4. doi: 10.1038/s41522-023-00474-5, 38238339 PMC10796366

[ref23] Li Z. Dong J. Wang M. Yan J. Hu Y. Liu Y. . (2022). Resveratrol ameliorates liver fibrosis induced by nonpathogenic Staphylococcus in BALB/c mice through inhibiting its growth. Mol. Med. 28:52. doi: 10.1186/s10020-022-00463-y, 35508992 PMC9066969

[ref24] Li S. Liu Z. Zhang Q. Su D. Wang P. Li Y. . (2024). The antidiabetic potential of probiotics: A review. Nutrients 16:2494. doi: 10.3390/nu16152494, 39125375 PMC11313988

[ref25] Li H. Lu W. Wang A. Jiang H. Lyu J. (2020). Changing epidemiology of chronic kidney disease as a result of type 2 diabetes mellitus from 1990 to 2017: estimates from global burden of disease 2017. J. Diabetes Investig. 12, 346–356. doi: 10.1111/jdi.13355, 32654341 PMC7926234

[ref26] Li L. Yang Y.n. Cao Y. Zhan J. Wu Y. Wu C. (2023). Perspective on the modern interpretation of the property theory of mild-natured and sweet-flavored traditional Chinese medicine via gut microbiota modulation. Integr. Med. Nephrol. Androl. 10:e00012. doi: 10.1097/imna-d-23-00012

[ref27] Liu E. Ji X. Zhou K. (2024). *Akkermansia muciniphila* for the prevention of type 2 diabetes and obesity: A meta-analysis of animal studies. Nutrients 16:3440. doi: 10.3390/nu16203440, 39458436 PMC11510203

[ref28] Mokdad A. H. Bisignano C. Hsu J. M. Ababneh H. S. Abbasgholizadeh R. Abdelkader A. . (2024). The burden of diseases, injuries, and risk factors by state in the USA, 1990–2021: a systematic analysis for the global burden of disease study 2021. Lancet 404, 2314–2340. doi: 10.1016/s0140-6736(24)01446-6, 39645376 PMC11694014

[ref29] Patil M. P. Ahire J. J. Patil U. K. Bhushan B. Chaudhari B. L. (2021). Effect of *Lactobacillus helveticus* CD6 on serum lipid profile and indicators of liver function in high-fat diet fed Swiss albino mice. 3 Biotech 11:266. doi: 10.1007/s13205-021-02832-6, 34017672 PMC8124034

[ref30] Qin L. Fan B. Zhou Y. Zheng J. Diao R. Wang F. . (2025). Targeted gut microbiome therapy: applications and prospects of probiotics, fecal microbiota transplantation and natural products in the management of type 2 diabetes. Pharmacol. Res. 213:107625. doi: 10.1016/j.phrs.2025.107625, 39875017

[ref31] Qu L. Ren J. L. Huang L. Pang B. Liu X. Liu X. D. . (2018). Antidiabetic effects of *Lactobacillus casei* fermented yogurt through reshaping gut microbiota structure in type 2 diabetic rats. J. Agric. Food Chem. 66, 12696–12705. doi: 10.1021/acs.jafc.8b04874, 30398060

[ref32] Su W. Yang Y. Zhao X. Cheng J. Li Y. Wu S. . (2024). Potential efficacy and mechanism of eight mild-natured and bitter-flavored TCMs based on gut microbiota: A review. Chin. Herb. Med. 16, 42–55. doi: 10.1016/j.chmed.2023.08.001, 38375054 PMC10874767

[ref33] Wang H. Li S. Zhang L. Zhang N. (2024). The role of fecal microbiota transplantation in type 2 diabetes mellitus treatment. Front. Endocrinol. 15:1469165. doi: 10.3389/fendo.2024.1469165, 39735647 PMC11671274

[ref34] Xin Y. Hu C. Li Y. Yang Z. Zhang L. Li A. . (2024). Immunomodulatory potential of *Lactobacillus helveticus* KLDS 1.8701 postbiotics: by regulating the Th17/Treg balance. Food Biosci. 61:104842. doi: 10.1016/j.fbio.2024.104842

[ref35] Xu W. Yu J. Yang Y. Li Z. Zhang Y. Zhang F. . (2023). Strain-level screening of human gut microbes identifies *Blautia producta* as a new anti-hyperlipidemic probiotic. Gut Microbes 15:2228045. doi: 10.1080/19490976.2023.2228045, 37408362 PMC10324434

[ref36] Yan S. Chen L. Li N. Wei X. Wang J. Dong W. . (2024). Effect of *Akkermansia muciniphila* on pancreatic islet β-cell function in rats with prediabetes mellitus induced by a high-fat diet. Bioresour. Bioprocess. 11:51. doi: 10.1186/s40643-024-00766-4, 38763955 PMC11102893

[ref37] Yang Y. Cheng J. Liu C. Zhang X. Ma N. Zhou Z. . (2024). Gut microbiota in women with polycystic ovary syndrome: an individual based analysis of publicly available data. EClinicalMedicine 77:102884. doi: 10.1016/j.eclinm.2024.102884, 39469535 PMC11513668

[ref38] Yang Y. Zhao X. Zou L. Lu W. Zhang X. Wu C. (2023). An analysis of gut bacterial and fungal community interactions in *Saxifraga stolonifera* Curt.-treated mice. Dis. Res. 3, 65–73. doi: 10.54457/dr.202302003

[ref39] Zhang C. Wang Z. Liu X. Liu X. Liu T. Feng Y. . (2024). *Akkermansia muciniphila* administration ameliorates streptozotocin-induced hyperglycemia and muscle atrophy by promoting IGF2 secretion from mouse intestine. iMeta 3:e237. doi: 10.1002/imt2.237, 39429872 PMC11487547

[ref40] Zhao X. G. Lin T. Jiang W. K. Lin Y. H. Xiao L. Y. Tian Y. F. . (2025). *Lactobacillus helveticus* LZ-R-5 ameliorates DSS-induced colitis in mice by modulating gut microbiota and enhancing intestinal barrier function. J. Agric. Food Chem. 73, 464–477. doi: 10.1021/acs.jafc.4c07895 39688942

[ref41] Zhong S. Yang Y.-N. Huo J.-X. Sun Y.-Q. Zhao H. Dong X.-T. . (2025). Cyanidin-3-rutinoside from Mori Fructus ameliorates dyslipidemia via modulating gut microbiota and lipid metabolism pathway. J. Nutr. Biochem. 137:109834. doi: 10.1016/j.jnutbio.2024.109834, 39694116

[ref42] Zhou D. X. Li S. J. Hu G. Wang Y. F. Qi Z. H. Xu X. . (2025). Hypoglycemic effect of *C. butyricum*-pMTL007-GLP-1 engineered probiotics on type 2 diabetes mellitus. Gut Microbes 17:2447814. doi: 10.1080/19490976.2024.2447814, 39745177 PMC12931707

[ref43] Zou L. E. Yang Y. N. Zhan J. Cheng J. Fu Y. Cao Y. . (2024). Gut microbiota-based discovery of Houttuyniae Herba as a novel prebiotic of *Bacteroides thetaiotaomicron* with anti-colitis activity. Biomed. Pharmacother. 172:116302. doi: 10.1016/j.biopha.2024.116302, 38387133

