# Correction: Novel probiotic *Lactobacillus helveticus* WIS02 alleviates diabetes through multi-pronged regulation of glycolipid metabolism, pancreatic protection and gut microbiota remodeling

**DOI:** 10.3389/fmicb.2026.1795126

**Published:** 2026-02-03

**Authors:** Shuang Guo, Yuying Li, Yanan Yang, Yuexiao Jiang, Yufeng Wang, Ying Cao, Yunfeng Duan, Chongming Wu

**Affiliations:** 1 School of Chinese Materia Medica, Tianjin University of Traditional Chinese Medicine, Tianjin, China; 2 PKUMed-Wisbiom Joint Laboratory for Human Microbiome Research, Beijing, China; 3 State Key Laboratory of Chinese Medicine Modernization, Tianjin, China; 4 Tianjin Key Laboratory of Therapeutic Substance of Traditional Chinese Medicine, Tianjin, China

**Keywords:** diabetes mellitus, gut microbiota, hypoglycemic effect, *Lactobacillus helveticus* WIS02, probiotic

A correction has been made to Section **3.3** ***Lactobacillus helveticus*** **QIS02 tends to favor the gut flora of diabetic animals**, **page 5, paragraph 2**. The sentence “At the phylum level, At the door level….” Is a duplicate.

The corrected sentence appears below:

“At the phylum level, the DM group showed elevated levels of *Proteobacteria* and reduced levels of *Firmicutes* and *Actinobacteria* compared to the normal group.”

A correction has been made to **4 Discussion**, **page 9, paragraph 3**. The sentence “Recent macro-genomics studies have revealed significant differences in the gut microbiota between healthy people and diabetic patients, suggesting that there is a significant association between the development of diabetes mellitus and the ecological dysregulation of gut microbiota [re].” contained a typo of the addition of “[re]”.

The corrected sentence appears below:

“Recent macro-genomics studies have revealed significant differences in the gut microbiota between healthy people and diabetic patients, suggesting that there is a significant association between the development of diabetes mellitus and the ecological dysregulation of gut microbiota.”

A correction has been made to Section **2.1 Preparation of bacterial suspensions, page 2, paragraph 1**.

The corrected sentence appears below:

“Glycerol-preserved *L. helveticus* WIS02(CGMCC No. 29243) was provided by MGBlab, Microbiota-Gut-Brain Laboratory, WISBIOM (Bejing) Biotechnology Co. Ltd., and was retrieved from ultra-low temperature storage, thawed at room temperature, and incoculated into de Man-Rogosa-Sharpe (MRS) broth for anaerobic recovery at 37 °C.”

A correction has been made to Section **3.2** ***Lactobacillus helveticus*** **WIS02 improves dyslipidemia in diabetic mice, page 5, paragraph 1**. There were duplicated descriptions.

The corrected sentence appears below:

“STZ induction significantly elevated serum TC, TG, and LDL-C in the DM group compared to Norm mice.”

A correction has been made to Section **3.3** ***Lactobacillus helveticus*** **WIS02 tends to favor the gut flora of diabetic animals, page 5, paragraph 3**. There was a misspelling of *Bacteroidetess*.

The corrected sentence appears below:

“On the contrary, *L. helveticus* WIS02 treatment significantly decreased the abundance of *Helicobacter cinaedi* and increased the abundance of *Bacteroidetes*, especially *Bacteroides caecimuris*.”

The original version of this article has been updated.

